# 4-Amino­pyridinium-3-sulfonate monohydrate

**DOI:** 10.1107/S1600536811002145

**Published:** 2011-01-22

**Authors:** Zhi-Biao Zhu, Shan Gao, Seik Weng Ng

**Affiliations:** a College of Chemistry and Materials Science, Heilongjiang University, Harbin 150080, People’s Republic of China; b Department of Chemistry, University of Malaya, 50603 Kuala Lumpur, Malaysia

## Abstract

The reaction of 4-amino­pyridine and oleum yielded the title hydrated zwitterion, C_5_H_6_N_2_O_3_S·H_2_O. There are two formula units in the asymmetric unit. The H and non-H atoms of both zwitterions lie on a mirror plane except for one sulfonate O atom. The water mol­ecules are also situated on a mirror plane. In the crystal, the zwitterions and water mol­ecules are linked by O—H⋯O and N—H⋯O hydrogen bonds, generating a three-dimensional network.

## Related literature

The analogous reaction of 4-hy­droxy­pyridine with oleum yielded hydro­nium 4-oxo-1,4-dihydro­pyridine-3-sulfonate dihydrate and 4-hy­droxy­pyridinium-3-sulfonate; see: Zhu *et al.* (2009 ▶, 2011 ▶).
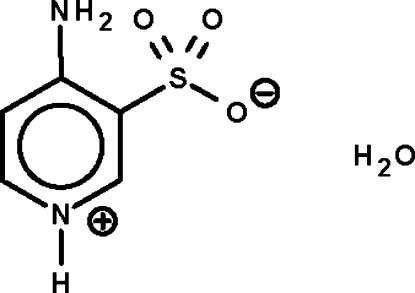


## Experimental

### 

#### Crystal data

C_5_H_6_N_2_O_3_S·H_2_O
*M* *_r_* = 192.19
Orthorhombic, 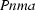

*a* = 31.6739 (13) Å
*b* = 6.5824 (3) Å
*c* = 7.3204 (3) Å
*V* = 1526.23 (11) Å^3^
*Z* = 8
Mo *K*α radiation
μ = 0.40 mm^−1^
*T* = 293 K
0.21 × 0.19 × 0.16 mm

#### Data collection

Rigaku R-AXIS RAPID diffractometer
Absorption correction: multi-scan (*ABSCOR*; Higashi, 1995 ▶) *T*_min_ = 0.921, *T*_max_ = 0.939
22965 measured reflections
1898 independent reflections
1325 reflections with *I* > 2σ(*I*)
*R*_int_ = 0.049

#### Refinement

*R*[*F* ^2^ > 2σ(*F* ^2^)] = 0.044
*wR*(*F* ^2^) = 0.137
*S* = 1.17
1898 reflections
157 parameters
8 restraints
All H-atom parameters refined
Δρ_max_ = 0.45 e Å^−3^
Δρ_min_ = −0.49 e Å^−3^

### 

Data collection: *RAPID-AUTO* (Rigaku, 1998 ▶); cell refinement: *RAPID-AUTO*; data reduction: *CrystalStructure* (Rigaku/MSC, 2002 ▶); program(s) used to solve structure: *SHELXS97* (Sheldrick, 2008 ▶); program(s) used to refine structure: *SHELXL97* (Sheldrick, 2008 ▶); molecular graphics: *X-SEED* (Barbour, 2001 ▶); software used to prepare material for publication: *publCIF* (Westrip, 2010 ▶).

## Supplementary Material

Crystal structure: contains datablocks global, I. DOI: 10.1107/S1600536811002145/im2250sup1.cif

Structure factors: contains datablocks I. DOI: 10.1107/S1600536811002145/im2250Isup2.hkl

Additional supplementary materials:  crystallographic information; 3D view; checkCIF report

## Figures and Tables

**Table 1:** Hydrogen-bond geometry (Å, °)

*D*—H⋯*A*	*D*—H	H⋯*A*	*D*⋯*A*	*D*—H⋯*A*
N1—H1⋯O1w^i^	0.88 (2)	1.99 (2)	2.820 (4)	158 (3)
N2—H22⋯O2^ii^	0.88 (2)	2.01 (2)	2.886 (3)	172 (3)
N3—H3⋯O1w^iii^	0.88 (2)	2.15 (3)	2.871 (4)	139 (3)
N4—H42⋯O4^iv^	0.88 (2)	1.99 (2)	2.869 (3)	173 (4)
O1w—H1w⋯O1	0.84 (2)	1.99 (2)	2.826 (3)	173 (3)
O2w—H2w⋯O3	0.85 (2)	2.02 (2)	2.864 (2)	171 (3)

Symmetry codes: (i) 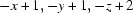; (ii) 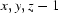; (iii) 
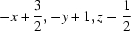
; (iv) 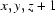.

**Fig. 1.**
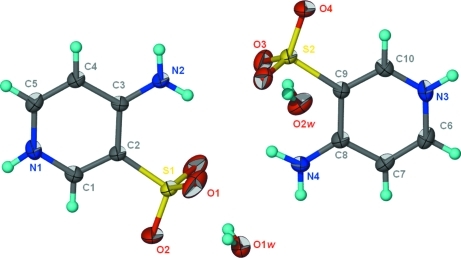
Thermal ellipsoid plot (Barbour, 2001) of the two independent molecules of C5H6N2O3S.H2O at the 50% probability level; hydrogen atoms are drawn as spheres of arbitrary radius.

## supplementary crystallographic information

### Comment

A previous reaction of 4-hydroxpyridine and oleum gave the salt, hydronium 4-oxo-1,4-dihydropyridine-3-sulfonate dihydrate (Zhu *et al.*, 2009); a later repeat of the synthesis gave zwitterionic 4-hydroxypyridinium-3-sulfonate (Zhu *et al.*, 2011). The studies were extended to 4-aminopyridine which upon reaction with oleum gave zwitterionic 4-aminopyridinium-3-sulfonate as a monohydrate (Scheme I, Fig. 1). The bonds in the ring are delocalized bonds. Adjacent zwitterions and water molecues are linked by N–H···O and O–H···O hydrogen bonds into a three-dimensional network (Tablel 1).

### Experimental

4-Aminopyridine (10 mmol) was dissolved in 20% oleum (10 ml). The solution was heated to 393 K for 4 days. After it was cooled, the excess oleum was decanted. Recrystallization of the solid from water gave colorless crystals.

### Refinement

Hydrogen atoms were placed in calculated positions (C–H 0.93 Å) and were included in the refinement in the riding model approximation, with *U*(H) set to 1.2*U*_eq_(C). The amino and water H atoms were located in a difference Fourier map, and were refined with distance restraints of O–H 0.84±0.01 Å and N–H 0.88±0.01 Å; their temperature factors were tied by a factor of 1.2–1.5*U*_eq_(N,O).

### Crystal data

**Table:**

C_5_H_6_N_2_O_3_S·H_2_O	*F*(000) = 800
*M**_r_* = 192.19	*D*_x_ = 1.673 Mg m^−^^3^
Orthorhombic, *P**n**m**a*	Mo *K*α radiation, λ = 0.71073 Å
Hall symbol: -P 2ac 2n	Cell parameters from 13913 reflections
*a* = 31.6739 (13) Å	θ = 3.1–27.5°
*b* = 6.5824 (3) Å	µ = 0.40 mm^−^^1^
*c* = 7.3204 (3) Å	*T* = 293 K
*V* = 1526.23 (11) Å^3^	Prism, colorless
*Z* = 8	0.21 × 0.19 × 0.16 mm

### Data collection

**Table:**

Rigaku R-AXIS RAPID diffractometer	1898 independent reflections
Radiation source: fine-focus sealed tube	1325 reflections with *I* > 2σ(*I*)
graphite	*R*_int_ = 0.049
Detector resolution: 10.000 pixels mm^-1^	θ_max_ = 27.5°, θ_min_ = 3.1°
ω scans	*h* = −41→41
Absorption correction: multi-scan (*ABSCOR*; Higashi, 1995)	*k* = −8→8
*T*_min_ = 0.921, *T*_max_ = 0.939	*l* = −9→8
22965 measured reflections	

### Refinement

**Table:**

Refinement on *F*^2^	Primary atom site location: structure-invariant direct methods
Least-squares matrix: full	Secondary atom site location: difference Fourier map
*R*[*F*^2^ > 2σ(*F*^2^)] = 0.044	Hydrogen site location: inferred from neighbouring sites
*wR*(*F*^2^) = 0.137	All H-atom parameters refined
*S* = 1.17	*w* = 1/[σ^2^(*F*_o_^2^) + (0.0795*P*)^2^] where *P* = (*F*_o_^2^ + 2*F*_c_^2^)/3
1898 reflections	(Δ/σ)_max_ = 0.001
157 parameters	Δρ_max_ = 0.45 e Å^−^^3^
8 restraints	Δρ_min_ = −0.49 e Å^−^^3^

### Fractional atomic coordinates and isotropic or equivalent isotropic displacement parameters (Å^2^)

**Table:**

	*x*	*y*	*z*	*U*_iso_*/*U*_eq_	
S1	0.55812 (2)	0.2500	0.86929 (9)	0.0348 (3)	
S2	0.66689 (2)	0.2500	0.36003 (10)	0.0360 (3)	
O1	0.58140 (6)	0.4293 (3)	0.8206 (3)	0.0647 (6)	
O2	0.54212 (8)	0.2500	1.0518 (3)	0.0712 (10)	
O1W	0.62156 (8)	0.7500	1.0095 (3)	0.0451 (6)	
H1W	0.6107 (9)	0.648 (4)	0.959 (4)	0.068*	
O2W	0.67561 (10)	−0.2500	0.6401 (4)	0.0573 (8)	
H2W	0.6649 (10)	−0.151 (4)	0.582 (4)	0.086*	
O3	0.64409 (5)	0.0686 (3)	0.4112 (2)	0.0496 (5)	
O4	0.68205 (8)	0.2500	0.1750 (3)	0.0570 (8)	
N1	0.43845 (8)	0.2500	0.7058 (4)	0.0413 (7)	
H1	0.4151 (7)	0.2500	0.770 (4)	0.050*	
N2	0.55273 (8)	0.2500	0.4434 (3)	0.0366 (7)	
H21	0.5763 (6)	0.2500	0.505 (4)	0.044*	
H22	0.5522 (11)	0.2500	0.3234 (14)	0.044*	
N3	0.78781 (8)	0.2500	0.5065 (4)	0.0416 (7)	
H3	0.8121 (6)	0.2500	0.449 (4)	0.050*	
N4	0.67536 (8)	0.2500	0.7841 (4)	0.0443 (8)	
H41	0.6504 (6)	0.2500	0.731 (5)	0.053*	
H42	0.6752 (12)	0.2500	0.9046 (15)	0.053*	
C1	0.47379 (9)	0.2500	0.8053 (4)	0.0367 (8)	
H1A	0.4717	0.2500	0.9320	0.044*	
C2	0.51300 (9)	0.2500	0.7265 (4)	0.0296 (7)	
C3	0.51604 (9)	0.2500	0.5320 (4)	0.0285 (7)	
C4	0.47767 (9)	0.2500	0.4337 (4)	0.0330 (7)	
H4	0.4783	0.2500	0.3067	0.040*	
C5	0.44023 (11)	0.2500	0.5209 (4)	0.0408 (8)	
H5	0.4153	0.2500	0.4535	0.049*	
C6	0.78747 (10)	0.2500	0.6914 (5)	0.0449 (9)	
H6	0.8129	0.2500	0.7549	0.054*	
C7	0.75061 (10)	0.2500	0.7851 (4)	0.0399 (8)	
H7	0.7510	0.2500	0.9121	0.048*	
C8	0.71128 (9)	0.2500	0.6911 (4)	0.0344 (7)	
C9	0.71321 (10)	0.2500	0.4964 (4)	0.0332 (7)	
C10	0.75175 (10)	0.2500	0.4121 (4)	0.0368 (8)	
H10	0.7529	0.2500	0.2852	0.044*	

### Atomic displacement parameters (Å^2^)

**Table:**

	*U*^11^	*U*^22^	*U*^33^	*U*^12^	*U*^13^	*U*^23^
S1	0.0304 (4)	0.0518 (6)	0.0223 (4)	0.000	−0.0034 (3)	0.000
S2	0.0287 (4)	0.0574 (6)	0.0219 (4)	0.000	−0.0017 (3)	0.000
O1	0.0602 (11)	0.0694 (15)	0.0644 (12)	−0.0289 (10)	−0.0289 (10)	0.0189 (10)
O2	0.0466 (15)	0.144 (3)	0.0225 (12)	0.000	−0.0016 (11)	0.000
O1W	0.0354 (13)	0.0573 (18)	0.0428 (14)	0.000	0.0008 (10)	0.000
O2W	0.0606 (18)	0.065 (2)	0.0457 (16)	0.000	−0.0179 (12)	0.000
O3	0.0365 (9)	0.0631 (13)	0.0493 (10)	−0.0121 (9)	−0.0081 (7)	0.0079 (9)
O4	0.0438 (14)	0.104 (2)	0.0237 (11)	0.000	−0.0003 (10)	0.000
N1	0.0255 (13)	0.059 (2)	0.0388 (15)	0.000	0.0049 (11)	0.000
N2	0.0287 (13)	0.0584 (19)	0.0227 (12)	0.000	−0.0021 (11)	0.000
N3	0.0240 (13)	0.055 (2)	0.0452 (16)	0.000	0.0040 (11)	0.000
N4	0.0337 (15)	0.076 (2)	0.0228 (13)	0.000	0.0004 (11)	0.000
C1	0.0335 (16)	0.050 (2)	0.0265 (14)	0.000	0.0026 (13)	0.000
C2	0.0280 (14)	0.0379 (19)	0.0227 (14)	0.000	−0.0013 (11)	0.000
C3	0.0303 (15)	0.0324 (18)	0.0227 (13)	0.000	0.0013 (11)	0.000
C4	0.0311 (15)	0.041 (2)	0.0267 (14)	0.000	−0.0032 (12)	0.000
C5	0.0325 (16)	0.054 (2)	0.0363 (17)	0.000	−0.0078 (13)	0.000
C6	0.0307 (16)	0.058 (2)	0.0461 (18)	0.000	−0.0103 (15)	0.000
C7	0.0372 (17)	0.052 (2)	0.0304 (16)	0.000	−0.0086 (14)	0.000
C8	0.0314 (16)	0.045 (2)	0.0270 (14)	0.000	−0.0013 (12)	0.000
C9	0.0279 (15)	0.046 (2)	0.0261 (14)	0.000	0.0004 (11)	0.000
C10	0.0333 (16)	0.045 (2)	0.0322 (16)	0.000	0.0038 (13)	0.000

### Geometric parameters (Å, °)

**Table:**

S1—O2	1.429 (2)	N3—H3	0.88 (2)
S1—O1	1.436 (2)	N4—C8	1.326 (4)
S1—O1^i^	1.436 (2)	N4—H41	0.88 (2)
S1—C2	1.771 (3)	N4—H42	0.88(2
S2—O4	1.437 (2)	C1—C2	1.369 (4)
S2—O3	1.4451 (18)	C1—H1A	0.9300
S2—O3^i^	1.4451 (18)	C2—C3	1.427 (4)
S2—C9	1.775 (3)	C3—C4	1.412 (4)
O1W—H1W	0.84 (3)	C4—C5	1.347 (4)
O2W—H2W	0.85 (2)	C4—H4	0.9300
N1—C1	1.335 (4)	C5—H5	0.9300
N1—C5	1.355 (4)	C6—C7	1.354 (5)
N1—H1	0.878 (10)	C6—H6	0.9300
N2—C3	1.331 (4)	C7—C8	1.423 (4)
N2—H21	0.87 (2)	C7—H7	0.9300
N2—H22	0.88 (2)	C8—C9	1.427 (4)
N3—C10	1.335 (4)	C9—C10	1.368 (4)
N3—C6	1.354 (4)	C10—H10	0.9300
			
O2—S1—O1	114.46 (10)	C1—C2—C3	118.8 (3)
O2—S1—O1^i^	114.46 (10)	C1—C2—S1	118.9 (2)
O1—S1—O1^i^	110.5 (2)	C3—C2—S1	122.3 (2)
O2—S1—C2	105.40 (14)	N2—C3—C4	120.2 (2)
O1—S1—C2	105.54 (9)	N2—C3—C2	123.0 (3)
O1^i^—S1—C2	105.54 (9)	C4—C3—C2	116.8 (3)
O4—S2—O3	114.29 (9)	C5—C4—C3	121.1 (3)
O4—S2—O3^i^	114.29 (9)	C5—C4—H4	119.5
O3—S2—O3^i^	111.47 (16)	C3—C4—H4	119.5
O4—S2—C9	104.71 (14)	C4—C5—N1	120.7 (3)
O3—S2—C9	105.51 (8)	C4—C5—H5	119.7
O3^i^—S2—C9	105.51 (8)	N1—C5—H5	119.7
C1—N1—C5	120.7 (3)	N3—C6—C7	120.9 (3)
C1—N1—H1	114 (2)	N3—C6—H6	119.6
C5—N1—H1	125 (2)	C7—C6—H6	119.6
C3—N2—H21	120 (2)	C6—C7—C8	120.7 (3)
C3—N2—H22	118 (2)	C6—C7—H7	119.7
H21—N2—H22	122 (3)	C8—C7—H7	119.7
C10—N3—C6	120.7 (3)	N4—C8—C7	120.2 (3)
C10—N3—H3	120 (2)	N4—C8—C9	123.4 (3)
C6—N3—H3	119 (2)	C7—C8—C9	116.4 (3)
C8—N4—H41	123 (3)	C10—C9—C8	119.3 (3)
C8—N4—H42	121 (3)	C10—C9—S2	119.0 (2)
H41—N4—H42	116 (4)	C8—C9—S2	121.8 (2)
N1—C1—C2	122.0 (3)	N3—C10—C9	122.0 (3)
N1—C1—H1A	119.0	N3—C10—H10	119.0
C2—C1—H1A	119.0	C9—C10—H10	119.0
			
C5—N1—C1—C2	0.0	C10—N3—C6—C7	0.0
N1—C1—C2—C3	0.0	N3—C6—C7—C8	0.0
N1—C1—C2—S1	180.0	C6—C7—C8—N4	180.0
O2—S1—C2—C1	0.0	C6—C7—C8—C9	0.0
O1—S1—C2—C1	−121.49 (11)	N4—C8—C9—C10	180.0
O1^i^—S1—C2—C1	121.49 (11)	C7—C8—C9—C10	0.0
O2—S1—C2—C3	180.0	N4—C8—C9—S2	0.0
O1—S1—C2—C3	58.51 (11)	C7—C8—C9—S2	180.0
O1^i^—S1—C2—C3	−58.51 (11)	O4—S2—C9—C10	0.0
C1—C2—C3—N2	180.0	O3—S2—C9—C10	120.95 (9)
S1—C2—C3—N2	0.0	O3^i^—S2—C9—C10	−120.95 (9)
C1—C2—C3—C4	0.0	O4—S2—C9—C8	180.0
S1—C2—C3—C4	180.0	O3—S2—C9—C8	−59.05 (9)
N2—C3—C4—C5	180.0	O3^i^—S2—C9—C8	59.05 (9)
C2—C3—C4—C5	0.0	C6—N3—C10—C9	0.0
C3—C4—C5—N1	0.0	C8—C9—C10—N3	0.0
C1—N1—C5—C4	0.0	S2—C9—C10—N3	180.0

Symmetry codes: (i) *x*, −*y*+1/2, *z*.

### Hydrogen-bond geometry (Å, °)

**Table:**

*D*—H···*A*	*D*—H	H···*A*	*D*···*A*	*D*—H···*A*
N1—H1···O1w^ii^	0.88 (2)	1.99 (2)	2.820 (4)	158 (3)
N2—H22···O2^iii^	0.88 (2)	2.01 (2)	2.886 (3)	172 (3)
N3—H3···O1w^iv^	0.88 (2)	2.15 (3)	2.871 (4)	139 (3)
N4—H42···O4^v^	0.88 (2)	1.99 (2)	2.869 (3)	173 (4)
O1w—H1w···O1	0.84 (2)	1.99 (2)	2.826 (3)	173 (3)
O2w—H2w···O3	0.85 (2)	2.02 (2)	2.864 (2)	171 (3)

Symmetry codes: (ii) −*x*+1, −*y*+1, −*z*+2; (iii) *x*, *y*, *z*−1; (iv) −*x*+3/2, −*y*+1, *z*−1/2; (v) *x*, *y*, *z*+1.

## References

[bb1] Barbour, L. J. (2001). *J. Supramol. Chem.* **1**, 189–191.

[bb2] Higashi, T. (1995). *ABSCOR* Rigaku Corporation, Tokyo, Japan.

[bb3] Rigaku (1998). *RAPID-AUTO* Rigaku Corporation, Tokyo, Japan.

[bb4] Rigaku/MSC (2002). *CrystalStructure* Rigaku/MSC, The Woodlands, Texas, USA.

[bb5] Sheldrick, G. M. (2008). *Acta Cryst.* A**64**, 112–122. 10.1107/S0108767307043930 18156677

[bb6] Westrip, S. P. (2010). *J. Appl. Cryst.* **43**, 920–925.

[bb7] Zhu, Z.-B., Gao, S. & Ng, S. W. (2009). *Acta Cryst.* E**65**, o2687. 10.1107/S1600536809040641 PMC2971452 21578293

[bb8] Zhu, Z.-B., Gao, S. & Ng, S. W. (2011). *Acta Cryst.* E**67**, o11. 10.1107/S1600536810049603 PMC3050178 21522621

